# Characterisation of a micrometer-scale active plasmonic element by means of complementary computational and experimental methods

**DOI:** 10.3762/bjnano.14.12

**Published:** 2023-01-16

**Authors:** Ciarán Barron, Giulia Di Fazio, Samuel Kenny, Silas O’Toole, Robin O’Reilly, Dominic Zerulla

**Affiliations:** 1 School of Physics, University College Dublin, Belfield, Dublin 4, Ireland

**Keywords:** active plasmonics, atomic force microscope, scanning Joule expansion microscopy (SJEM), surface plasmon polariton

## Abstract

In this article, we investigate an active plasmonic element which will act as the key building block for future photonic devices. This element operates by modulating optical constants in a localised fashion, thereby providing an external control over the strength of the electromagnetic near field above the element as well as its far-field response. A dual experimental approach is employed in tandem with computational methods to characterise the response of this system. First, an enhanced surface plasmon resonance experiment in a classical Kretschmann configuration is used to measure the changes in the reflectivity induced by an alternating electric current. A lock-in amplifier is used to extract the dynamic changes in the far-field reflectivity resulting from Joule heating. A clear modulation of the materials’ optical constants can be inferred from the changed reflectivity, which is highly sensitive and dependent on the input current. The changed electrical permittivity of the active element is due to Joule heating. Second, the resulting expansion of the metallic element is measured using scanning Joule expansion microscopy. The localised temperature distribution, and hence information about the localisation of the modulation of the optical constants of the system, can be extracted using this technique. Both optical and thermal data are used to inform detailed finite element method simulations for verification and to predict system responses allowing for enhanced design choices to maximise modulation depth and localisation.

## Introduction

Active plasmonics has been gaining attention from the research community for its role in the development of photonic devices [1–2], low-loss waveguides [3], and imaging systems [4]. It is an emerging subfield of plasmonics, which focuses on controlling electromagnetic fields at the nanoscale through external manipulation of the materials’ properties. Here, we present the characterisation of a recently developed active plasmonic element [5] through two complementary experimental methods. Active plasmonic elements have applications in future imaging technologies and as modulators in optoelectronic couplers for photonic circuits. Finite element method (FEM) simulations are used to validate both experimental approaches, allowing for cross-verification of results and giving greater insight into the underlying physical phenomena.

Surface plasmon polaritons (SPPs) are mixed states of photons and electron density waves propagating along the interface between a conductor and a dielectric. As a result of this phenomenon, an electric field strongly confined in the *z*-direction is produced at the interface. As direct excitation of a smooth metallic surface does not form SPPs, certain configurations have been developed to provide the conditions allowing for its formation. These were initially proposed by Otto [6] and Kretschmann and Raether [7]. To meet momentum-matching conditions necessary for the formation of SPPs, such configurations rely on the presence of an optically denser dielectric material with which the light interacts before reaching the metal. Light–matter interactions which give rise to the formation of SPPs can be classified into a sub-field of photonics known as plasmonics [8]. Investigations into SPPs provide vital insights into fundamental physical phenomena at the nano- and mesoscales [9–15], as well as more practical applications in Raman spectroscopy in the form of surface-enhanced Raman spectroscopy (SERS) [16] and other spectroscopic techniques [17–18]. SPPs also find uses in fields such as ultrasensitive detection methods [19–20], as their formation is highly dependent on refractive index changes, and sub-wavelength optics [21]. Our active plasmonic element also provides the potential for an even more sensitive technique. Active plasmonics has further advantages due to the tunable nature of the physics underneath and its ability to interact with electronic circuits [22].

The performance of the active element can be characterised in terms of modulation localisation and depth. Localisation addresses how confined the active control is at the nanoscale, while modulation depth is an indicator of how well the external manipulation changes the properties of the device. Characterisation of both localised temperature distribution and optical constants plays a key role for further applications and is required to optimise the operation parameters for the active plasmonic element.

Previous studies [23–24] investigated the effects of gap size using a fine tunable mechanical separation as a means to control the intensity of a travelling SPP on silver. In contrast, in the present work, the modulation of the device’s response is obtained through changes in the optical constants via electrical signals. It is well understood that heating affects the electrical permittivity of metals [25–28] and dielectrics [29–30]. This, in conjunction with Joule heating, is used to generate the desired effects.

**Figure 1:**
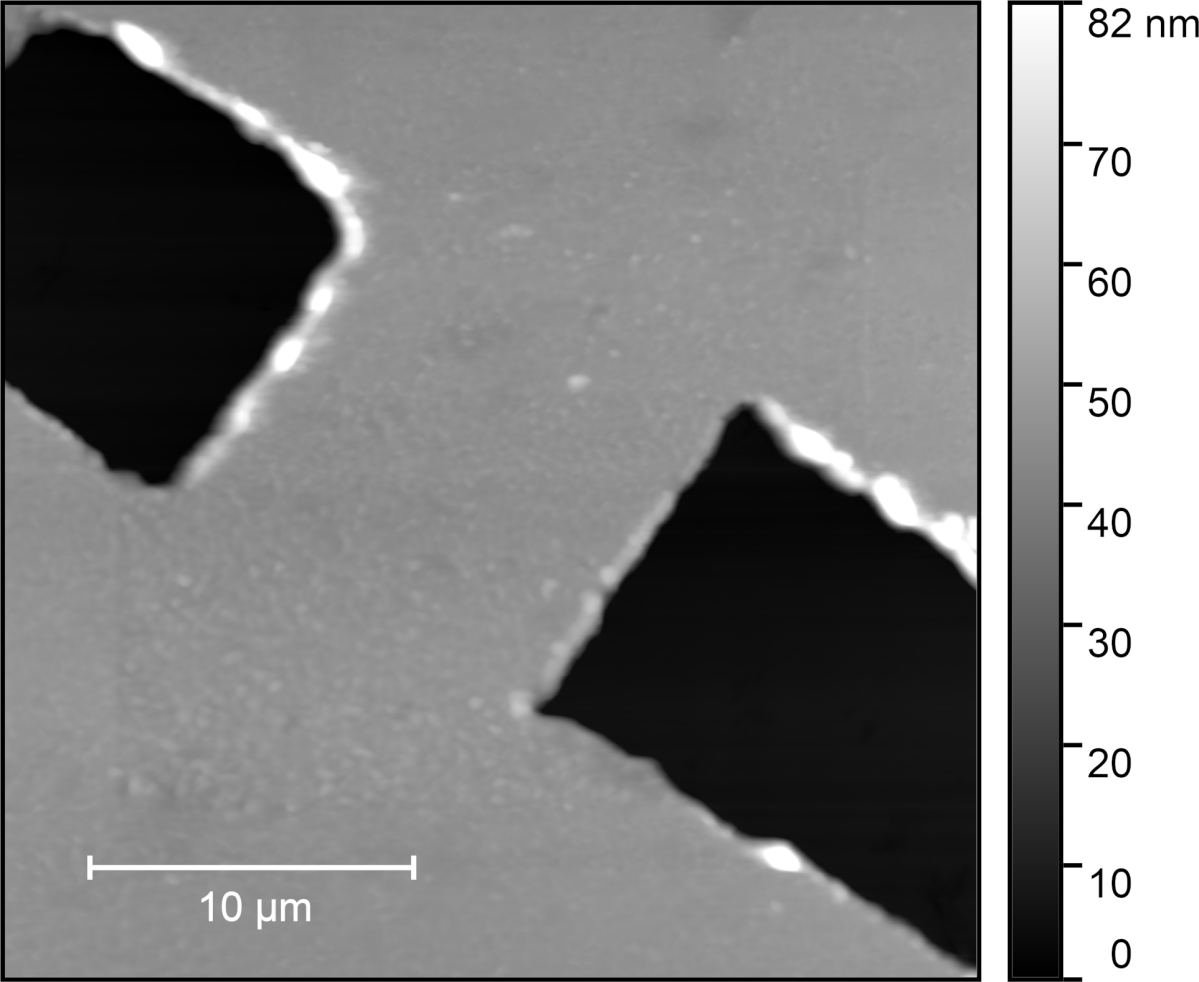
An AFM image of a 10 × 10 μm^2^ constriction in a 48 nm thin silver film on a sapphire substrate. The total area of the AFM image is 30 × 30 μm^2^. Two cuts (black channels) split the silver layer (grey) into two parts, connected only by the bridge depicted in the image. Under the application of a current, the metal heats up due to the Joule effect. The presence of a constriction in the metal (the bridge) results in a localised heating effect.

The active plasmonic element proposed (Figure 1) consists of a nano- or mesoscale constriction in a 48 nm thick layer of silver. Applying a current through the silver layer results in increased heating at the constriction due to the reduced cross section. Consequently, given the dependence of the materials electric permittivity on temperature, the optical response will change locally.

In this work, we have adapted two unconventional correlative methods to investigate the system exploiting their ability to directly probe the parameters of interest. The first method is based on an attenuated total reflection (ATR) setup whose versatility is enhanced through the addition of a lock-in amplifier (LIA) investigating the changes in the reflectivity induced by a modulated electric current. The acquisition of a surface plasmon resonance (SPR) curve is a common method to characterise a plasmonic far-field response [31] which is highly sensitive to small changes in the refractive index of the metal and dielectric. Because of this extreme sensitivity, small changes in the local temperature, and hence in optical constants, will result in subtle but appreciable changes of reflectivity in the SPR curve. Homodyne detection, with the modulated electric currents as reference, enables a detailed examination of the microscale active plasmonic element. Temperature changes are induced by the applied current through the microoptical element. Analysis of both the lock-in signal and the classical SPR curve from angular interrogation enables the deduction of the complex optical constants. Furthermore, the presence of strong peaks indicates the ability to maximise the modulation of the element’s optical response. While the classical SPR response integrates over the whole illuminated area, the LIA curve extracts the changes in reflectivity localised to the active element. This leads to a modulated local near field, which has applications in the development of new imaging technologies using this localised field to go beyond the diffraction limit of light. Because the temperature is geometry-dependent in the constriction, it is necessary to map the thermal distribution in the vicinity of the element. The experimental complementary method therefore investigates the thermal response of this active plasmonic element at a high spatial resolution. Knowledge of the distribution leads to predictions on how the near field will be locally affected, which is key for understanding the behaviour of the active plasmonic element. The heating distribution is investigated by means of scanning Joule expansion microscopy (SJEM) [32]. The technique provides a method to obtain the relative temperature distribution at the nanoscale starting from the measurement of induced thermal expansion, which can be directly mapped in a standard AFM-based image using a LIA. This also provides great insight into nanomechanical thermal interactions.

Knowledge of both physical parameters, the optical response, and heating allows cross-verification and understanding of the underlying mechanisms causing changes in the active plasmonic element. The FEM simulation software COMSOL Multiphysics has been used to predict and confirm the dynamic temperature distribution and the optical response of the system.

## Experimental

The manufacturing of the active plasmonic elements employed in the present work is detailed in [5]. At first, a 48 ± 2 nm film of silver is deposited on a sapphire substrate via physical vapour deposition (PVD). After this, two separate AFMs are used to machine channels in the silver film to create the desired constriction, which in this case measures 10 μm. The tip of the AFM is held at a set loading force in contact with the thin metal film and moved to remove the silver.

The investigations carried out consist of two experimental correlative methods. The first method is the above discussed enhanced SPR experiment, based on an ATR setup whose sensitivity is enhanced through the addition of a lock-in amplifier. The second method probes the temperature distribution surrounding the active element through SJEM mapping the thermal expansion of the metallic surface using an AFM. Both methods are further reinforced through the use of three-dimensional simulations. A description of the experimental methods of both investigations is detailed below as well as the FEM parameters used to simulate the behaviour of an active plasmonic element.

### Enhanced SPR experiment

**Figure 2:**
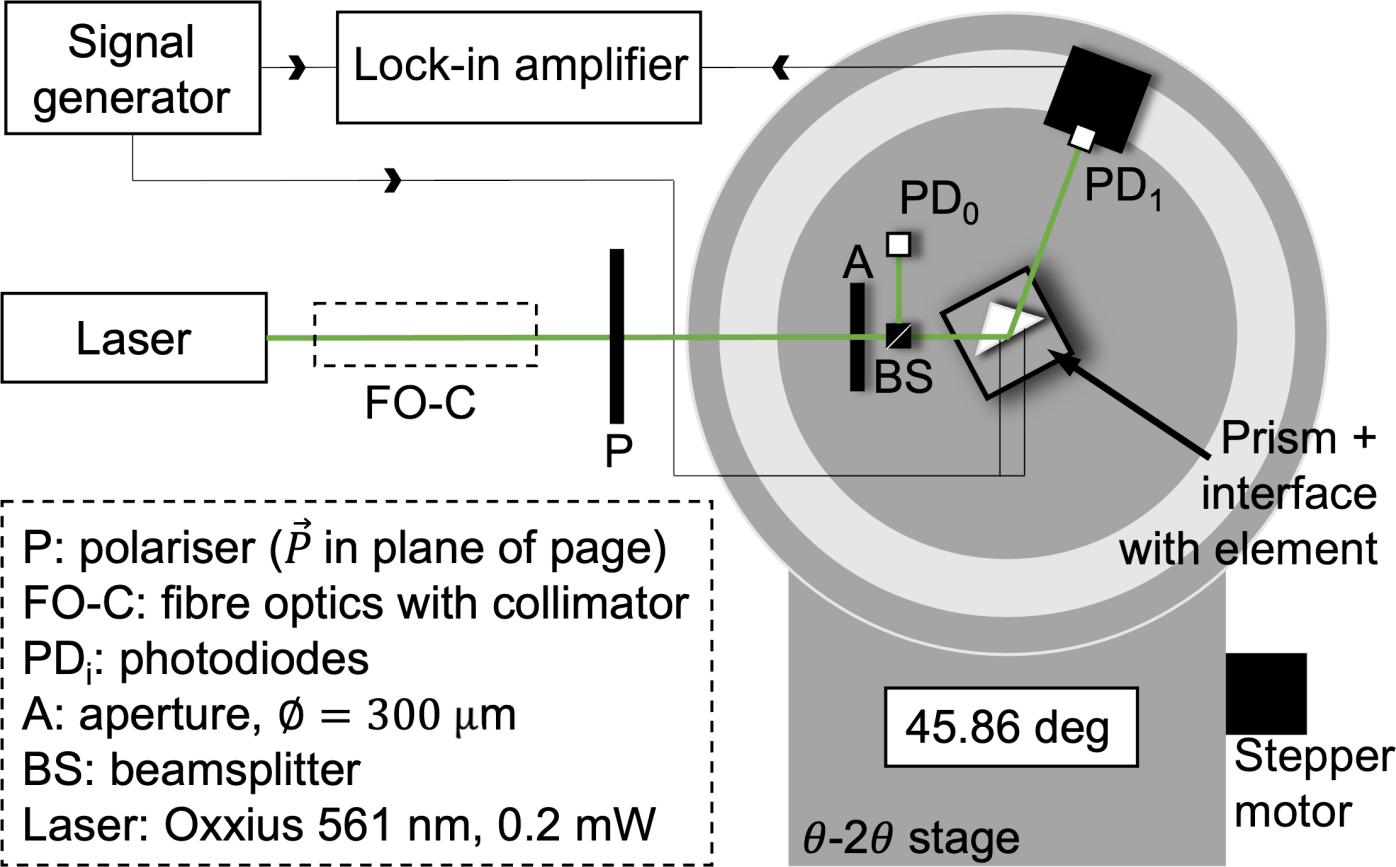
A schematic of the enhanced SPR experiment.

For angular interrogation of the plasmonic response of the active plasmonic element, an enhanced SPR experiment (Figure 2) was used. The system under investigation consists of a constriction in the silver film such that a current modulated at a particular frequency affects its optical properties via Joule heating. The signal is acquired by a photodiode and further processed by a lock-in amplifier (Ametek 5210), with the driving signal of the modulated voltage acting as reference. The DC component of the light results in a typical SPR curve while the modulated signal from the lock-in amplifier produces a more sensitive signal. This yields the variation in the optical constants as a function of the thermal modulation of the active element.

An Oxxius single-frequency CW laser at 561.4 nm, typically incident at 0.2 mW after filtering, was used as the incident light source. The collimated light from an optical fibre was spatially filtered through a 300 μm aperture and a polariser aligned to the plane of rotation ensuring *p*-polarisation for SPP excitation as seen in Figure 2. The reference light is recorded after the aperture and reflected from a cube beam splitter, with the signal photodiode placed on the 2θ arm of a high-accuracy (18 arcsec resolution) Siemens θ–2θ X-ray diffractometer stage with inbuilt goniometer to collect light reflected from the interface. The absolute angular position is manually determined by aligning multiple back reflections, with an estimated error of approximately 4.5 arcmin. The stage’s gearing is driven by a high-current pure sine signal such that motor steps cannot be missed, thus eliminating this as a source of error. The Kretschmann configuration was placed horizontally at the centre of the stage rotating at θ. This configuration consists of a fused silica prism with a sapphire slide optically coupled using refractive index matching oil (*n* = 1.516). This configuration was also used in the simulations. A sapphire slide was used as the deposition substrate for a thin silver film of 48 nm. The incident angles were referenced to the air–prism interface. The sinusoidal current was generated using a function generator with a current buffer to ensure impedance matching to the system under investigation. A transimpedance-amplified photodiode signal with active DC and high-frequency filtering was fed to the lock-in amplifier, while the non-filtered signal was recorded for the SPR reflected signal. The modulated voltage applied was of the same form as the simulations with frequency *f* = 631 Hz and offset such that the resultant signal was entirely positive.

At each angular position, the signals from the photodiodes were continuously recorded for one time constant following an adequate rest period, after which the in-phase components of the lock-in amplifier (*X*-components) were recorded using an Arduino-controlled analog-to-digital converter. The reference phase of the LIA was chosen to maximise *X*. The LIA was set to a time constant of τ = 300 ms, with second-order low-pass filtering and a sensitivity of 30 mV to avoid spurious input overloads. Once this set was recorded, the stage was moved 1 × 10^−2^ degrees to the next angular position in the scan. The substrate used to generate the SPR response is sapphire with a refractive index of *n* = 1.7717 at λ = 561 nm.

### SJEM experiment

To further characterise the active plasmonic element, in complement with the SPR curve measurements above, the thermal distribution due to Joule heating of the active element was measured through the use of scanning Joule expansion microscopy. The application of a current to the metallic element results in Joule heating. As stated previously, this heating results in an appreciable change in the optical constants of the silver enabling modulation of its plasmonic response. This heating also results in thermal expansion of the element. This expansion will result in deflection of an AFM cantilever scanning the surface. If a sinusoidal voltage is applied to an electrically conducting sample, such as the active plasmonic element discussed here, the resulting thermal expansion will be periodically modulated at the frequency of the applied voltage. When an AFM scan is performed on an element which is periodically modulated, the expanded surface will also be captured by the AFM. A lock-in amplifier can then be used to extract the periodically occurring expansion of the topography from a surface scan performed while the element is modulated at a known frequency. This is the basis for SJEM measurements.

**Figure 3:**
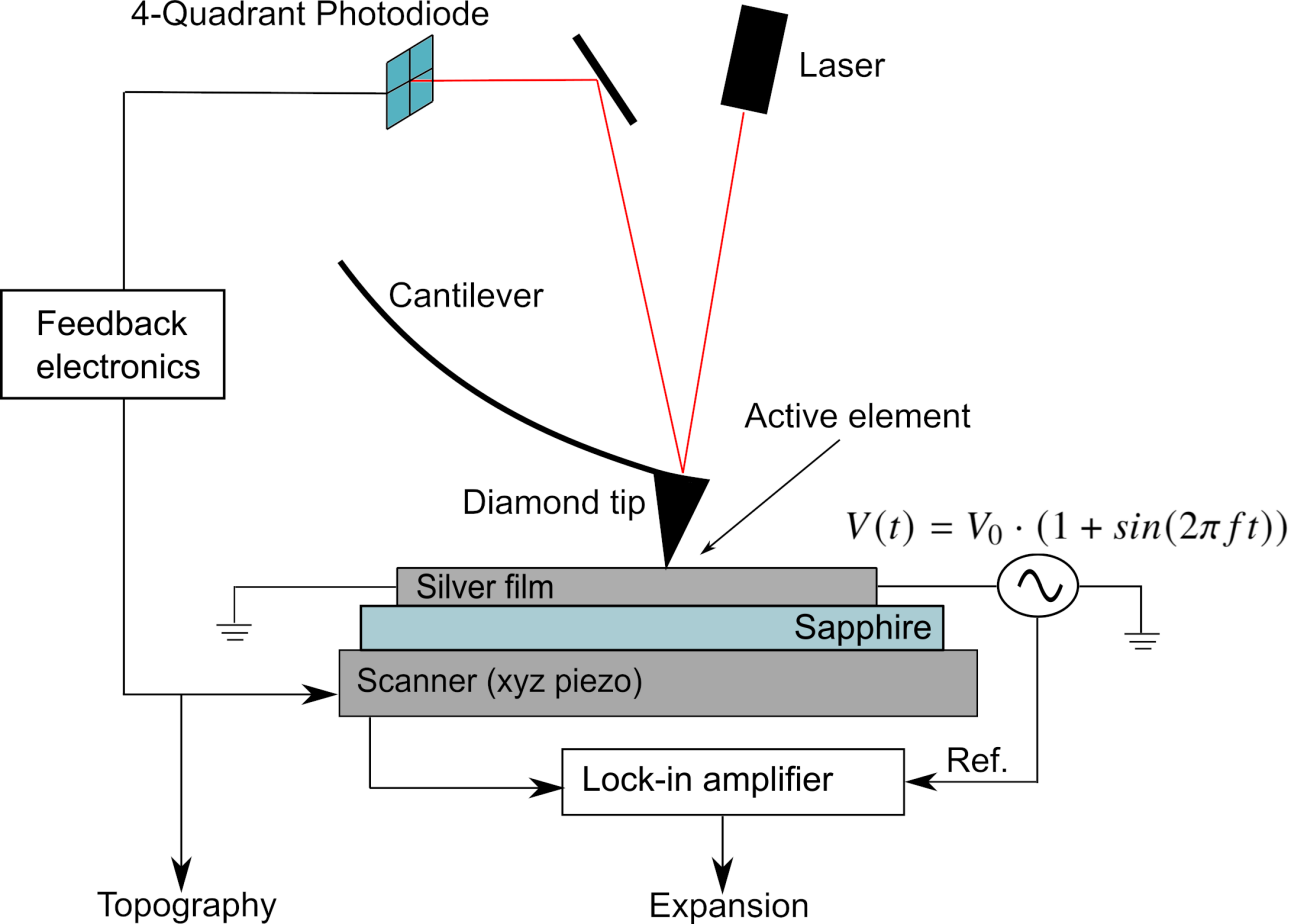
Schematic describing the experimental setup used to perform SJEM measurements on a modulated element.

Here, SJEM measurements have been performed using an Oxford instruments Cypher-S AFM and a signal recovery 7270 DSP lock-in amplifier. Figure 3 illustrates the setup used to perform such measurements. An Adama NM-RC probe (spring constant: 290.3 N/m, nominal resonance frequency: 814 kHz) has been used in contact mode to scan the topography of an electrically modulated sample with a loading force of 1.9 μN. This particular probe is intended for use in nanomechanical operations such as lithography and machining. The high spring constant of this cantilever has the advantage of minimising the unwanted deflection of the cantilever resulting from electrostatic interaction of the potential on the surface and the probe. The tip is constructed from wear-resistant diamond with a tip radius of 10 ± 5 nm. The deflection sensitivity of the probe was measured to be 81.09 nm/V.

To perform an SJEM measurement, a sinusoidal voltage is applied to the metallic element. The surface of the element is then scanned with an AFM in contact mode. Contact mode was selected in this case to ensure the probe captured the deflection due to thermal expansion while minimising artifacts caused by the periodic potential on the surface. The sample was driven with a frequency of 1227 Hz, well below the 170 kHz limitation on the response time of the *z*-piezo control of the scanner in the Cypher AFM. This frequency is also well below the resonance frequency of the selected cantilever avoiding unwanted resonant oscillations in the cantilever as a result of the periodic force applied by the expanding surface when deflecting the cantilever. In principle, the drive frequency of the active element could be the same for both experiments. However, the higher frequency provided an increased number of heating cycles over which to integrate given the chosen scan parameters listed below. Ideally, the active element would be modulated in the same fashion in both experiments. However, the information extracted in both cases does not depend on the drive frequency, given the thermal relaxation time of such a metallic element on a sapphire substrate is of the order of nanoseconds, and as such there is no need for matching drive frequencies. AFM scans were performed on a 30 × 30 μm^2^ window with a setting of 512 points per line. The resulting pixel size is 58.7 nm. AFM scans were then performed at a scan rate of 0.1 Hz resulting in the tip being over each pixel of the image for approximately 10 ms. At a drive frequency of 1227 Hz, this corresponds to more than ten cycles of expansion and contraction per point for the lock-in amplifier to integrate over. The time constant for the lock-in process was set to 10 ms to match the time the tip is over each pixel. The sensitivity of the LIA was set to 200 μV. The applied voltage was set based on the current density through the sample so as to match the conditions in the above SPR measurements as closely as possible. The Cypher-S AFM is capable of detecting dynamic changes in height of the sample down to the sub-picometer scale. Scans were performed at multiple current densities to demonstrate the differences in temperature distribution surrounding the sample as a function of applied current. For higher current densities, a wider distribution of the temperature is expected. A current buffer was used to ensure the current density through the element was consistent throughout an AFM scan. The probe was left electrically floating during scanning. While leaving the probe floating is counterproductive from the perspective of minimising electrostatic interaction, the possibility of current flow through the tip to ground is eliminated. As with the LIA phase selection for the SPR measurements discussed above, the phase was selected so as to maximise the *X*-component of the LIA signal. SJEM measurements were performed for current densities of 45, 48.2, 51.8, 54, and 58 mA/μm^2^, the results of which can be found below.

### Simulations

**Figure 4:**
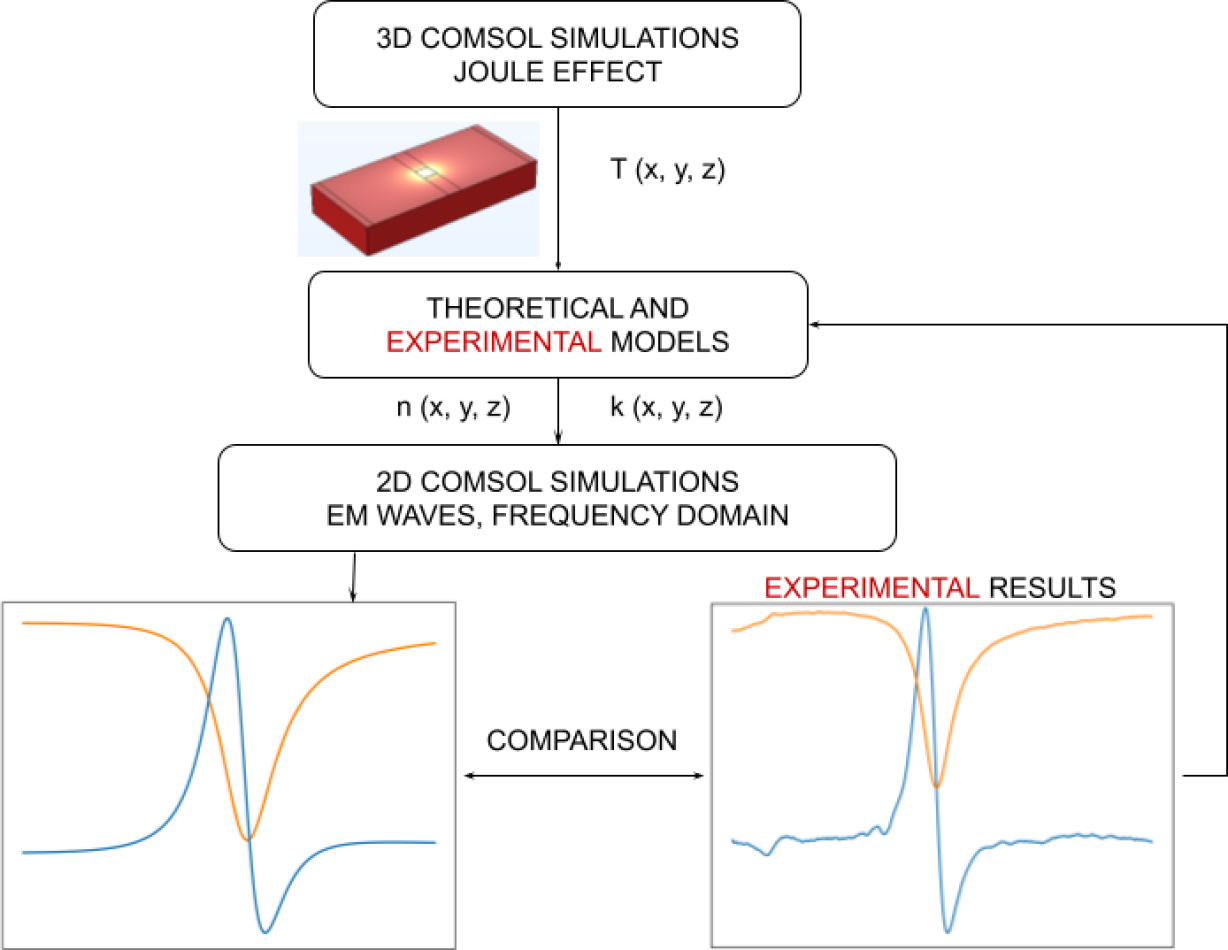
Flow diagram of simulation process.

Finite element analysis simulations using COMSOL Multiphysics 6.0 were employed to cross-verify the results obtained from both methods. The schematic in Figure 4 illustrates the steps involved in performing such simulations.

Initially, a model of the silver structure described above was built on top of a sapphire substrate. This model is a representative subsection of the substrate and silver film on which the physical active element was fabricated. The thermal behaviour of the element was simulated for an applied alternating electrical potential with a DC offset through the thin silver layer and compared to experimental findings. A temperature boundary condition was imposed on both the bottom and lateral surfaces of the model, keeping them at room temperature (293.15 K).

An alternating voltage with a DC offset *V*_in_(*t*) = *V*_0_ · (1 + sin(2π*ft*)), where *V*_0_ = 200.0 mV and *f* = 631 Hz, was applied across the silver layer. The voltage was chosen so as to guarantee a correspondence with the experiments in terms of current density. The application of a voltage with an AC component resulted in a spatial temperature distribution changing periodically as a function of the time. The temperature distribution shown in the Results section below in Figure 12 refers to the point in time *t* = 1.68 ms. Results in terms of temperature distribution were therefore used to cross-verify the experimental findings coming from the SJEM experiment.

The second part of the computational work focused on the changes induced by the temperature on the optical properties of the system under analysis. Such changes lead to differences in the reflectivity curve acquired by means of the enhanced SPR experiment. In order to back up the experimental findings, electromagnetic simulations were performed both in a cold state, using room temperature refractive indices, and in a hot state. Johnson and Christy [33] electrical permittivity values were used to model the silver layer at room temperature. The electromagnetic simulations of the heated system were performed starting from a temperature distribution extracted from the thermal simulations previously performed (*t* = 1.68 ms). This means that a temperature value is associated to each spatial point. Starting from two tables, one for sapphire and one for silver, containing spatial coordinates and corresponding temperature values, the data illustrated in Winsemius et al. [27] were used to model the influence of temperature on the silver’s permittivity while for sapphire results from Thomas et al. [34] were employed. The resulting tables, again one for each material, containing the spatial points and refractive indices were subsequently imported into COMSOL to define the heated materials. All values were evaluated at the selected operational wavelength λ = 561 nm. For reference, in the following Table 1, we report the refractive index values for both sapphire and silver at room temperature and at the highest temperature reached by the simulated structure. The refractive index of air was taken as *n*_air_ = 1.00.

**Table 1:** Refractive indices for silver and sapphire at room temperature and at the highest temperature reached by the structure at time *t* = 1.68 ms.

	Sapphire		Silver	

temperature (K)	293.15	338.26	293.15	340.00
*n*	1.7697	1.7703	0.056299	0.060110
κ	0.0	0.0	3.6867	3.6868

**Figure 5:**
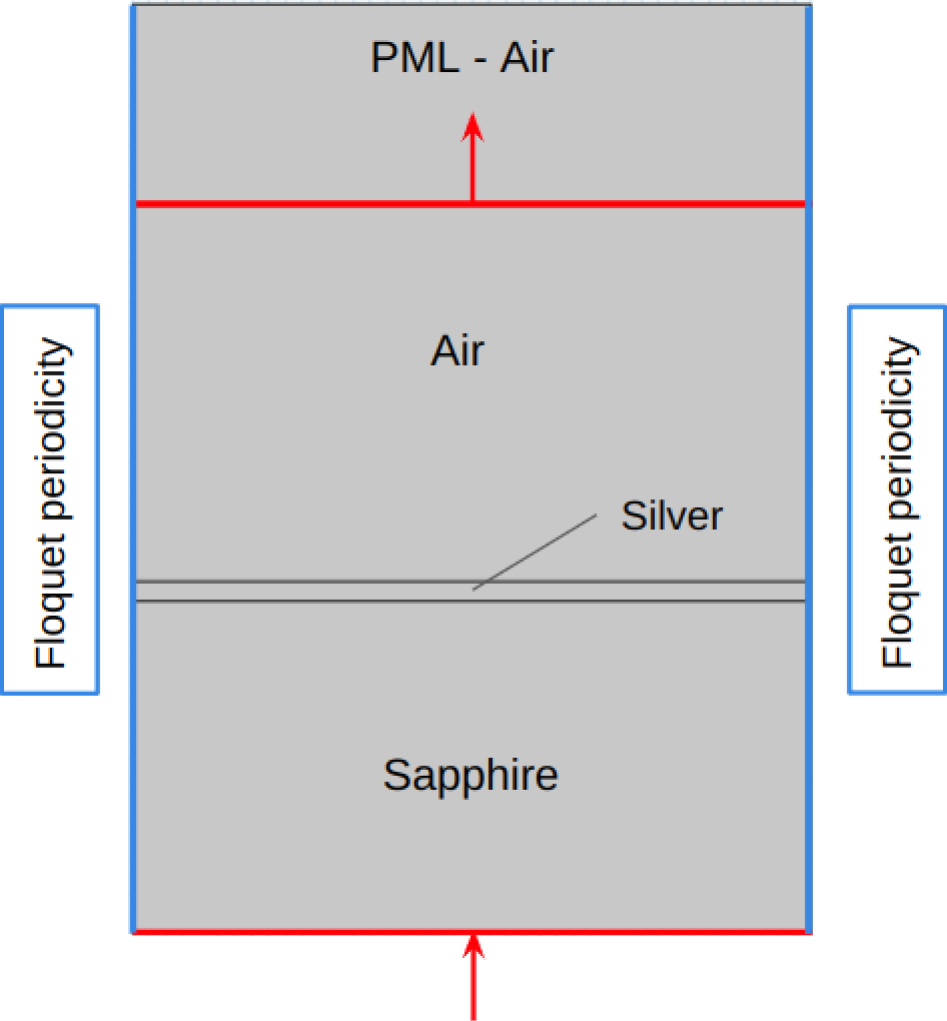
FEM optical simulation setup.

The optical response of the sapphire–metal–air structure was quantified in terms of reflectivity at different incoming excitation angles, computed by means of COMSOL optical simulations. A two-dimensional simulation space was set up in COMSOL, and the electromagnetic waves in the frequency domain module were used to set up the problem (see Figure 5). The input port was set to simulate an incoming TM wave, and Floquet periodic conditions were imposed on the side boundaries. A perfectly matched layer (PML) was added on top of the air layer in order to avoid back reflection. Total reflectivity data were collected at the output port.

Integrating this simulation setup with the temperature distribution output from the previous step allows for an accurate representation of temperature-dependent changes on the reflectivity curve. This produces a representation of the modulation depth which is seen when looking at the difference of hot and cold states using a lock-in amplifier.

## Results

First, the results of the SPR curve with the lock-in amplifier which measures the difference between a heated and room temperature constriction are presented. Subsequently, the results obtained from SJEM measurements are analysed. Finally, both experimental results are compared with simulations.

### Enhanced SPR experiment

**Figure 6:**
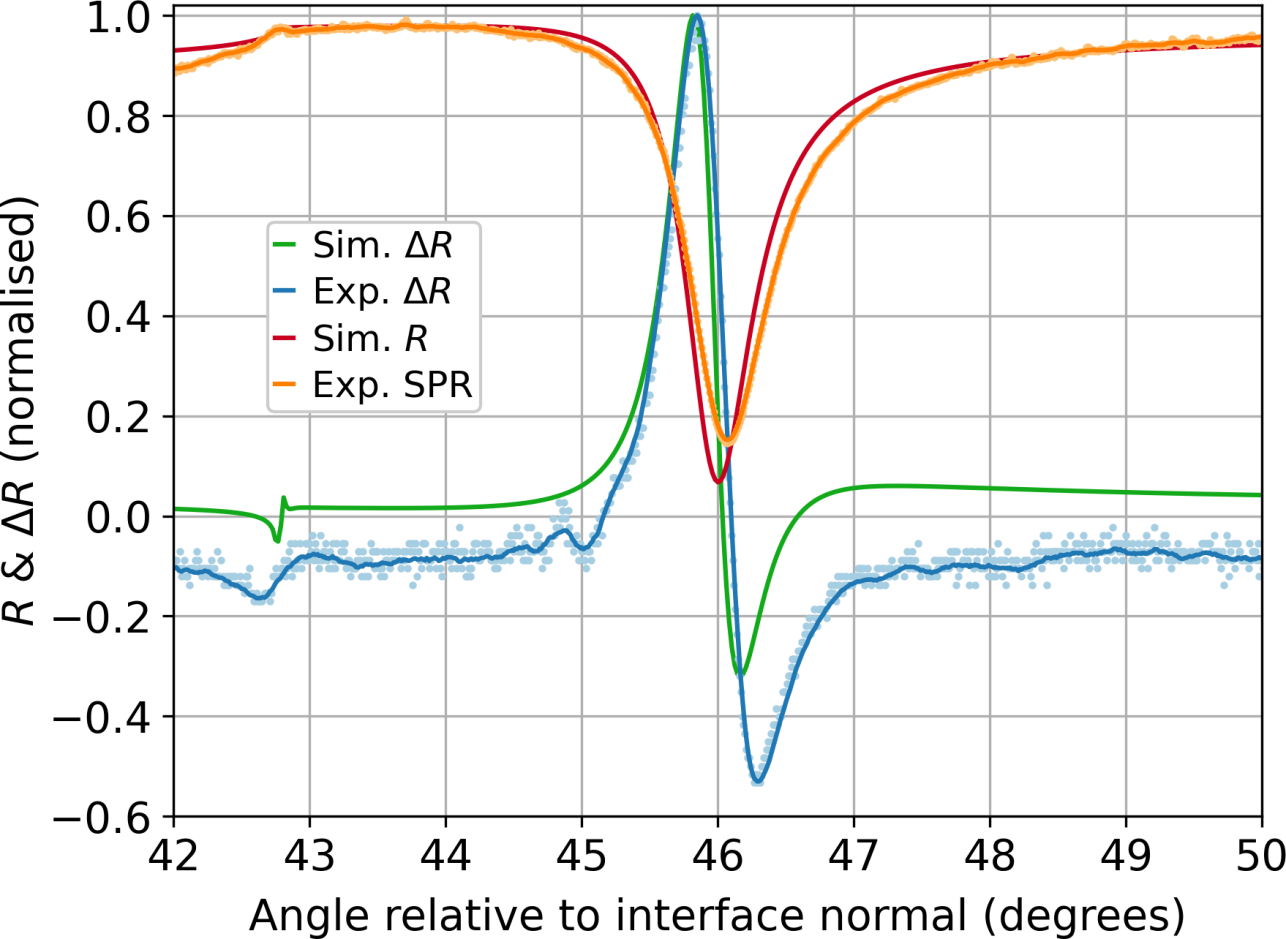
Plots of the enhanced SPR curves for a sapphire–silver–air configuration excited at 561 nm showing the typical shape in red and orange, and Δ*R* response, taken from the lock-in amplifier (*X*) component, or the effective difference between the coldest and hottest states in blue and green for a modulated 20 μm square active element. Orange shows the experimental curve normalised to the simulation maximum for an applied (RMS) current density of 71 mA/μm^2^. The angular position of the TIR response of the experimental SPR curves has been calibrated to the simulation. The solid lines are a centred moving average for each experimental curve. Experimental errors are considered below in Figure 8. The results of COMSOL 2D optical simulations were computed for a system at room temperature and with a temperature distribution extracted from thermal simulations. The curves presented are the average between those two states (red) and their difference (green).

Figure 6 shows the experimental data and simulated SPR curves. It is clear that all expected main features of the curves are present for both approaches. The SPP angle corresponds to a minimum in reflection and, in this case, was 46.07°. The results show a critical total internal reflection (TIR) angle at approximately 42.7° from the surface normal. Both results are in line with theoretical predictions for the employed substrate.

While the experimental measurements are exploiting a continuous sinusoidal modulation which oscillates between two states (i.e., hot and cold), the simulations are comparing only these extreme states. The simulation was performed on sapphire and silver at room temperature and at the heated state. In the experiment, the resulting SPR curve, recorded in parallel to the extracted lock-in curve, is effectively a weighted average of all temperature states. Therefore, the simulated SPR curves obtained from both the cold and the heated structure were averaged to obtain the red curve in Figure 6, and their relative difference was taken to generate the green curve.

The slight discrepancy between the minimum in the measured SPR curve (orange) and the theoretical version (red) is assumed to be ascribable to the data set used to model the simulated materials. To describe silver’s optical constants at room temperature, Johnson and Christy [33] data were employed, but the refractive index of the silver material used in the experiments differs slightly. The measured SPR curve is slightly broader than the simulated one. This is due to experimental broadening (in particular, the divergence of the laser beam), surface roughness, and further temperature-induced degradation effects that are not taken into account in the simplified simulation setup employed.

**Figure 7:**
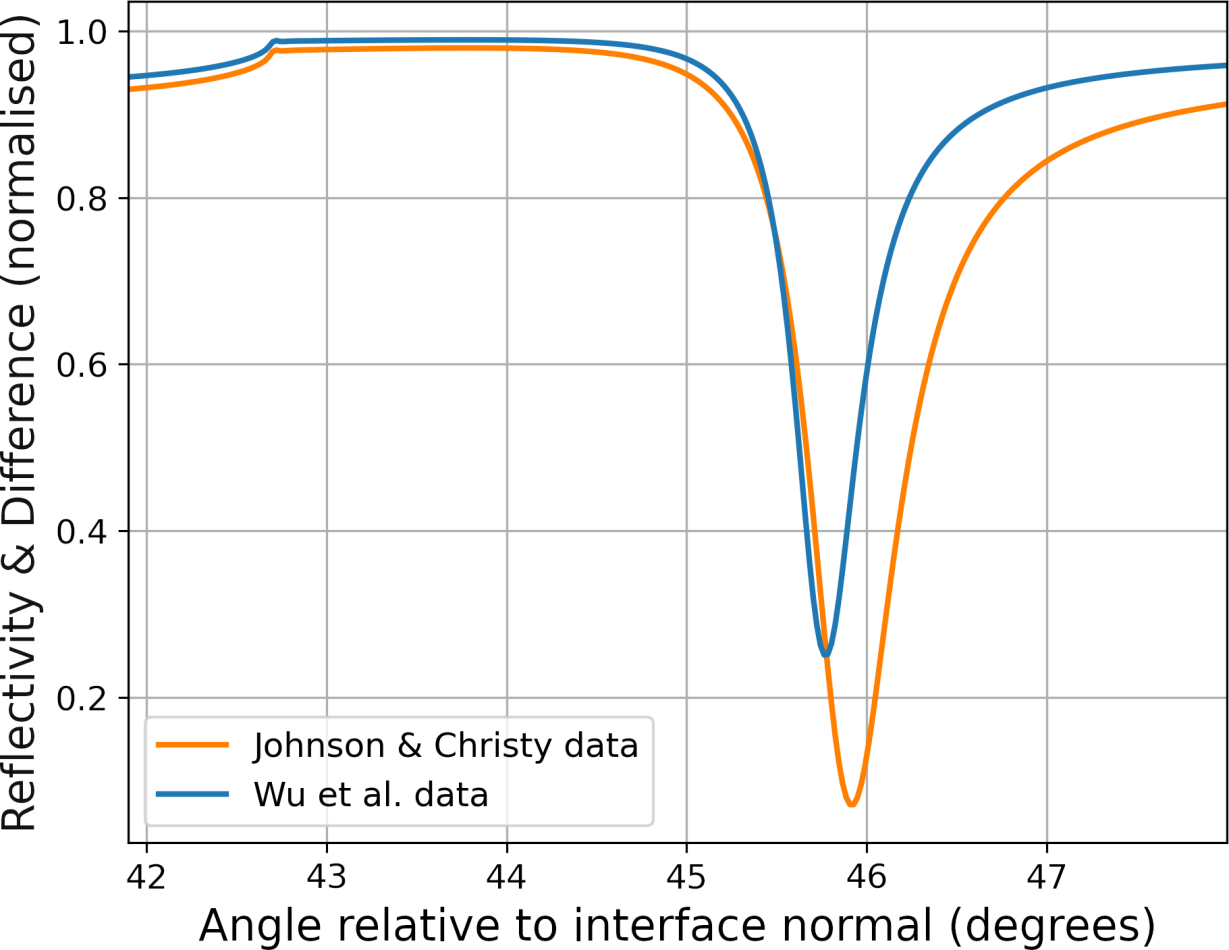
SPR curves resulting from electromagnetic simulations performed on a similar structure at room temperature. Different data sets were employed to model the silver layer. There is a noticeable shift in the position of the minimum of the two curves.

In order to determine the quality of the agreement between simulations and experimental data, we additionally carried out a comparative study between two electromagnetic simulations. Both simulations were set up at room temperature (293.15 K, cold state) and using the same substrate, but the silver layer was characterised in two different ways, using two of the most cited sources in the field. At first, the refractive index was defined using Johnson and Christy dataset [33]. Subsequently, the same simulation was repeated exploiting data from Wu and co-workers [35]. The result in terms of SPR curve is plotted in Figure 7.

Directly comparing the simulated and experimental plasmonic resonance positions between the SPR curves in Figure 6, there is a difference of 0.07° in the position of the plasmonic dip. While this is outside the estimated error accumulation of approximately 0.02° on the experimental data, the two simulations show a discrepancy of 0.14°. This clearly shows that the shift measured between the experimental SPR curve and the simulated one can be brought back to the difference between literature data for refractive indices and the optical properties of the film.

On close inspection of Figure 6, in contrast to simulations, the experimental measurements reveal additional peaks on the leading edge of the main peak about 45.0°. Detailed analyses reveal this is due to ageing of the silver and surface modifications. There are others at about 45.5°, which, altogether, will be discussed in a subsequent paper. At this stage, experiments with active elements of different sizes reveal that the ratio between heated and non-heated surface illuminated by the laser beam influences the intensity of this effect. The ability to observe these details demonstrates the high sensitivity of this technique to the differences in optical constants of small areas under investigation. This effect, while highly interesting, deserves its own dedicated investigation and, hence, falls outside the scope of this paper.

**Figure 8:**
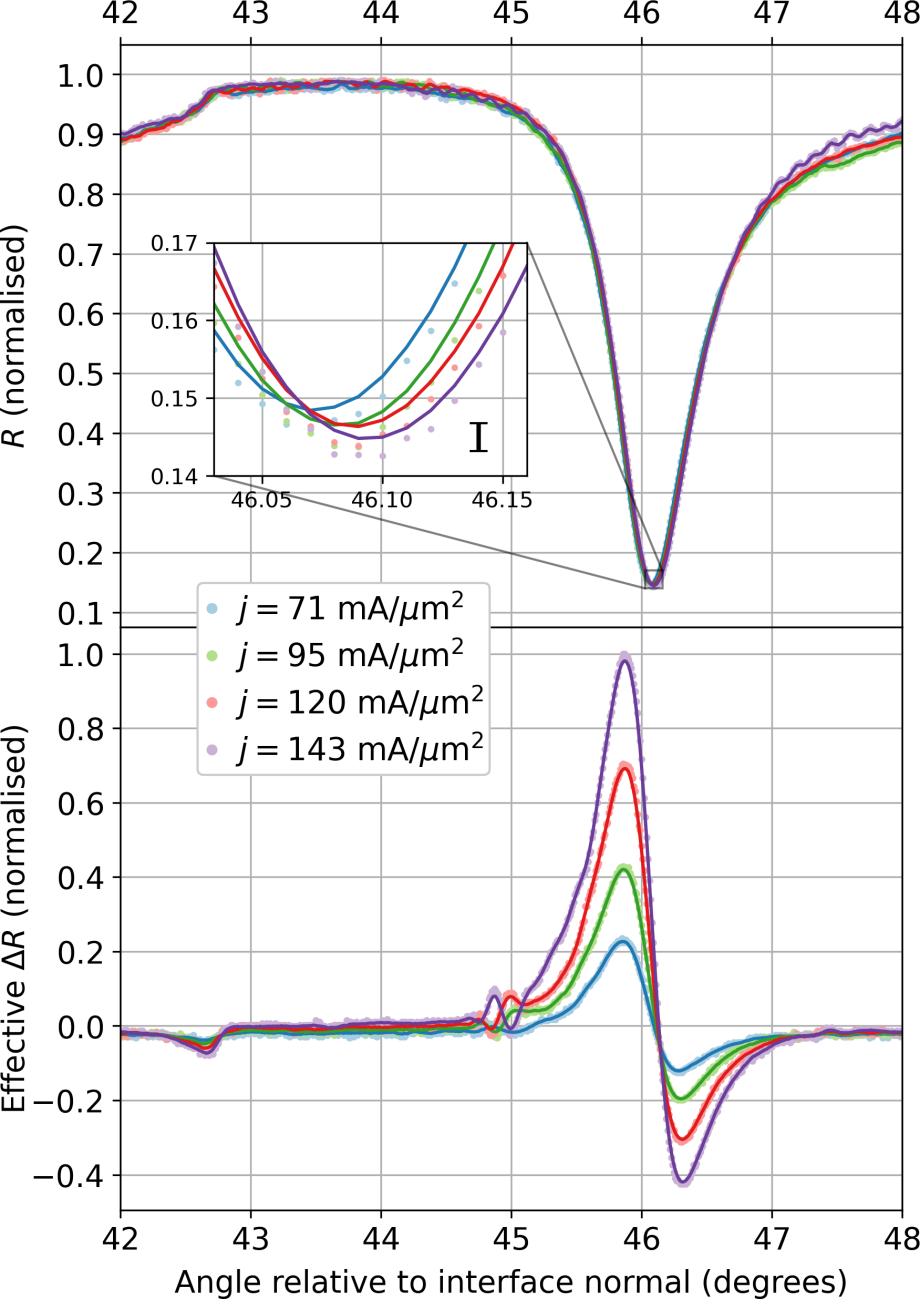
Plots of the enhanced SPR curves showing the typical SPR curve shapes (top) for a sapphire–silver–air configuration excited at 561 nm and the effective change in reflectivity (bottom) for a 20 μm square active element modulated with an applied (RMS) current density of 71–143 mA/μm^2^ in steps of ≈24 mA/μm^2^. The lock-in signal error is estimated at 0.5%. The inset emphasises the shift in the plasmonic resonance position for increasing temperatures with the error of the SPR signals included in black. The current densities were applied in ascending order. The solid lines are a centred moving average for each curve.

In Figure 8, the response of both the typical SPR curve and the LIA signal at four selected current densities and, hence, varied temperature, are shown. Current densities of 71–143 ± 1 mA/μm^2^ in steps of ≈24 mA/μm^2^ were used for a 20 μm square element. While not identical to the SJEM experimental current densities, we ensure that they are of similar magnitude, 45–58 ± 1 mA/μm^2^, hence, enabling comparison between the experiments. The increasing temperature is seen through the typical SPR curves as a broadening in the plasmonic dip and shift of the minimum angle towards higher angles, as expected.

Experimentally, the SPR curve is measured directly by the photodiode. The modulation depth of the reflected light selected here, in this simple geometry, is of the order of 1% with respect to the total light intensity. To improve the signal-to-noise ratio, a lock-in amplifier is used to detect modulated signals referenced at the driving frequency. The aforementioned SPR characteristics manifest in the *X*-component signal of the lock-in amplifier, which originates from the changes in the temperature and is highly sensitive to a modification of the systems morphology with a fixed phase relationship.

As the temperature is modulated, these alterations occur in tandem with the driving frequency. The increased temperature affects a change in the optical constants of the system, thereby altering the SPR curve including its resonance position. The difference is measured directly with the driving signal as reference using a lock-in amplifier. This in-phase signal of the LIA is a direct measurement of the changes in the SPR curve.

In conclusion, both the ATR response and its characteristic plasmonic dip in reflectivity are present in the simulated curve and their angular position show a very good agreement with experimental results. The relative difference between the two curves behaves in line with the observed lock-in signal, as reported in Figure 6, validating the measurements.

### SJEM experiment

**Figure 9:**
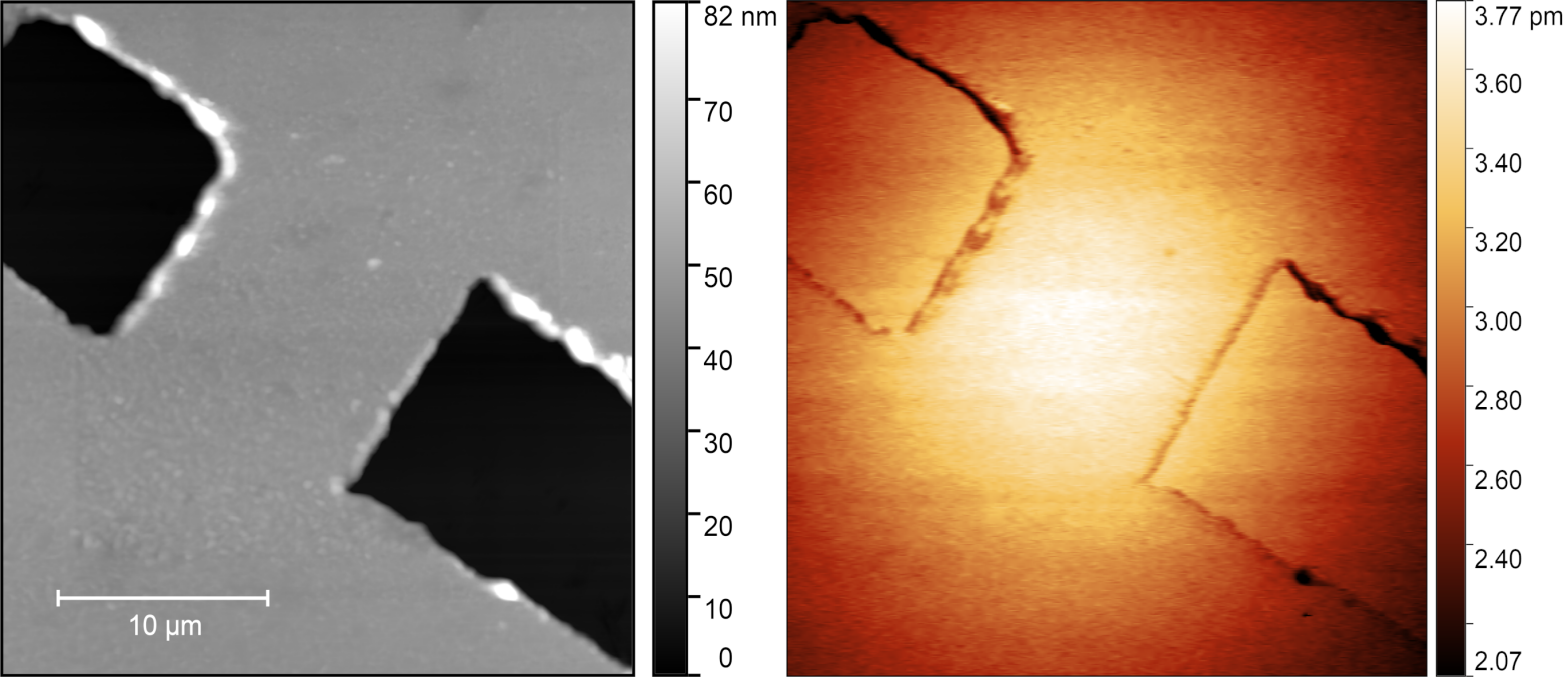
Left: Topography of the active element. AFM scan of 30 × 30 μm^2^ area of a 10 μm wide constriction in a 48 nm thin silver film. Right: Expansion of active element due to applied voltage V(*t*), as defined above, for a current density of 44 mA/μm^2^. Both scans are 512 × 512 pixels yielding a spatial step size of 58.7 nm.

Figure 9 shows an AFM image of the silver surface in a 30 × 30 μm^2^ window centered on the active element. On the left is a topographical image of the surface showing the element, which consists of a constriction of approximately 10 × 10 μm^2^ in the silver film. The accompanying colour bar denotes the height of the sample in reference to the sapphire substrate below. The dark regions are areas where the silver has been removed as described above. On the right is an SJEM measurement showing the thermal distribution around the active element by mapping the thermal expansion of the silver element for each pixel of the image. The SJEM image shows the largest expansion occurring at the centre of the element, decreasing away from the centre of the element approximating a Voigt function. The associated colour bar shows the highest expansion as lighter coloured regions and areas of lower expansion as darker regions. The expansion around the element is representative of the temperature distribution in the heated state of modulation and appears to follow the expected spread.

**Figure 10:**
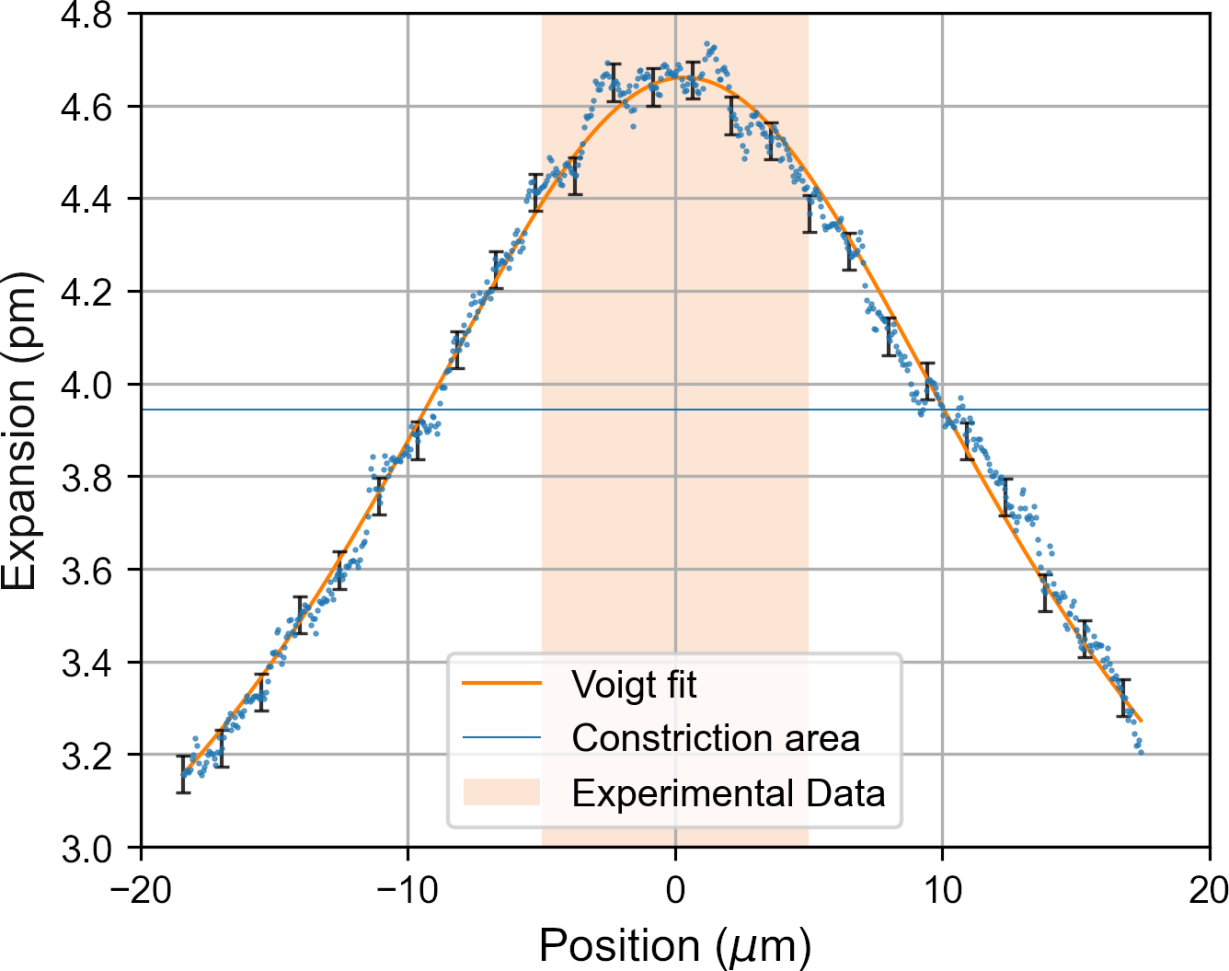
Experimental data showing the expansion profile for a 45.0 mA/μm^2^ modulation and relative fitting with a Voigt function. The shaded area corresponds to the surface of the constriction. The position along *x* was shifted so to have the distribution centered around 0 for visualisation purposes. Estimated errors on the SJEM measurement are shown every twenty-fifth data point.

**Figure 11:**
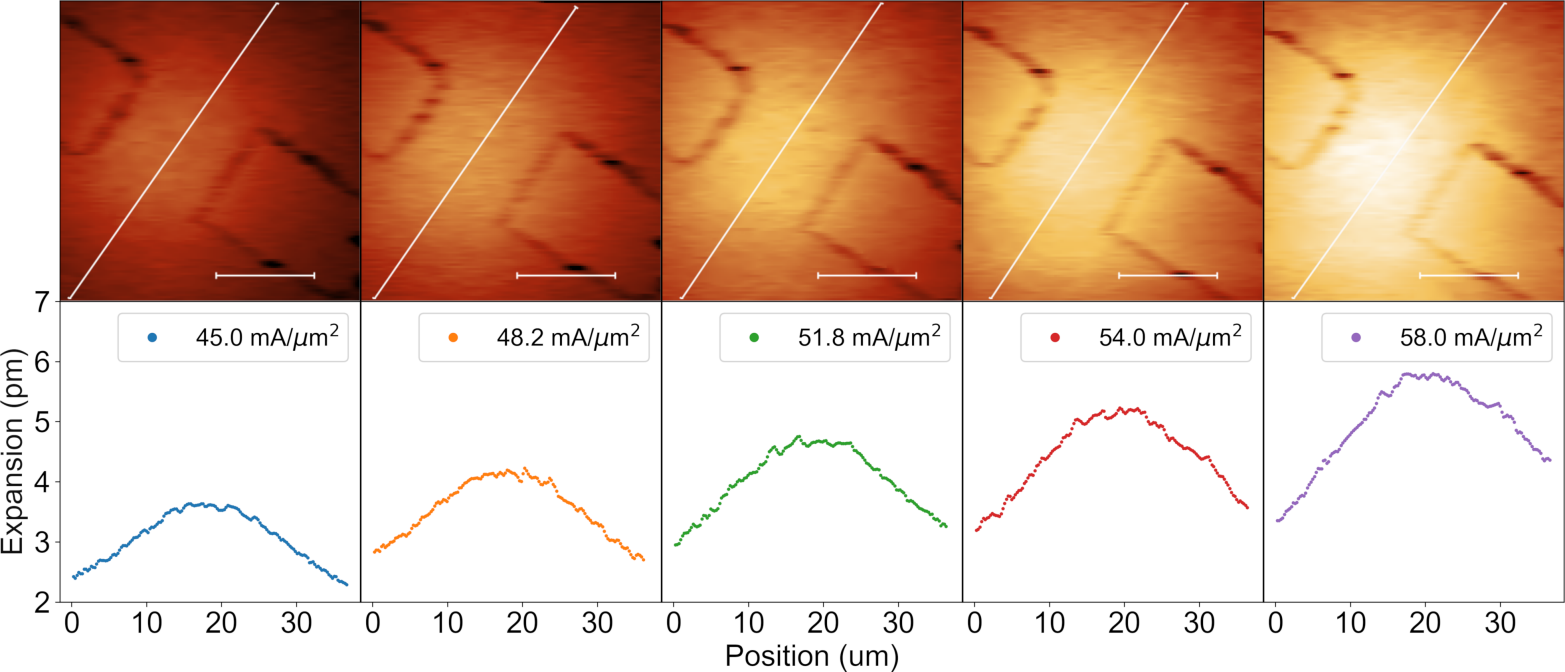
Thermal expansion of active element at varied current densities of 45.0, 48.2, 51.8, 54.0, and 58.0 mA/μm^2^. Accompanying line profiles show expansion along the centre, represented by white lines shown in the SJEM images, of the element. The scale bars provided on the SJEM Images are 10 μm wide. Errors are assumed to be the same as shown in Figure 10 for all measurements shown.

Figure 10 shows a Voigt function fit to a profile along the centre of the expansion image seen in Figure 9. The measured data is well represented by this fit function. The data shown in Figure 10 corresponds to the profile shown on the bottom left of Figure 11. The data has been translated along the *x*-direction so as to centre the peak of the fit at 0 μm for the purposes of visualisation. The area of the element is shown by the shaded region labelled “Constriction area” in Figure 10. From this shaded region we can see that the Voigt fit is centered slightly to the side of the centre of the element. This difference has been attributed to asymmetries in the fabricated structure resulting from the machining process [5].

As with the enhanced SPR measurements above, the effect of varying current has been investigated through repeated SJEM measurements under differing applied *V*(*t*). Figure 11 shows the results of these measurements. The images at the top show the spatially resolved thermal expansion across the active plasmonic element. The plots below show line profiles through the centre of the element at each current density. The shared *y*-axis allows for the comparison of expansion values measured at each current density. As the current density increases, the expansion of the element is seen to increase. This is in line with expectations as the temperature of the element should increase with increasing current density through the element. It should be noted that the positive *x*-direction here is moving from top to bottom along the profiles shown on the SJEM images in Figure 11. As expected, raising the current causes an expansion of the element in each case. Additionally, the thermal distribution broadens as the temperature of the element increases. The following detailed FEM simulations were used to verify the above results and can additionally be utilised to extract absolute temperatures from the dynamic SJEM measurements.

**Figure 12:**
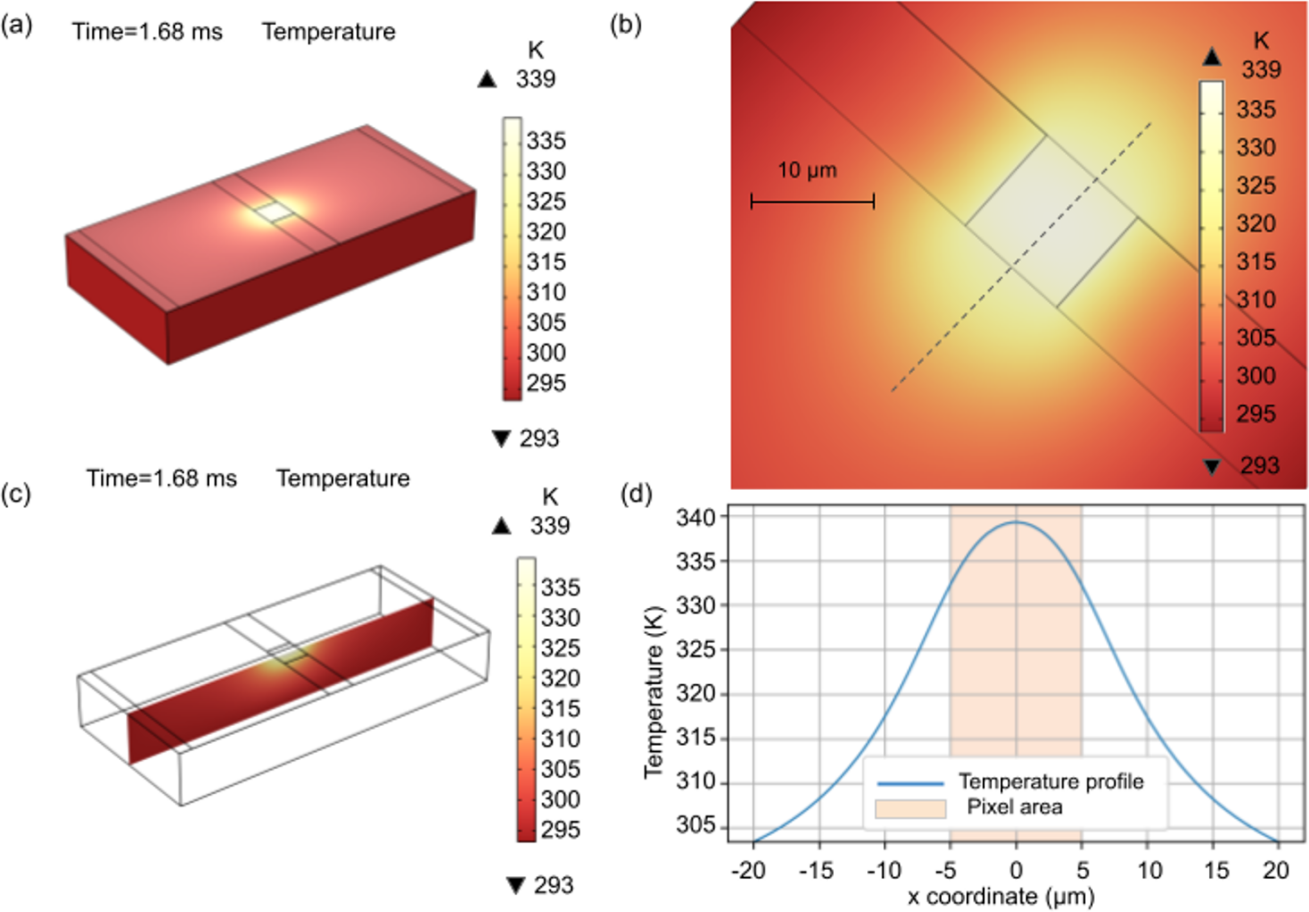
Results of thermoelectrical simulations done with COMSOL. (a) Surface temperature distribution for the whole structure and (b) in the neighbourhood of the constriction. (c) Temperature distribution on the *xz*-surface at *y* = 0 and (d) temperature distribution profile along the *x*-direction.

In Figure 12, we see the thermal effects of the modulated current across the structure under analysis simulated through COMSOL Multiphysics. As described in the first panel, the highest temperature region is clearly localised in the neighbourhood of the restriction, and the distribution spreads across the surrounding area following a profile with a FWHM ≈17.7 μm. This is more clearly depicted in Figure 12d, illustrating the temperature profile as a function of the *x*-coordinate. For comparison, Figure 10 shows an equivalent profile obtained experimentally for a 10 × 10 μm^2^ structure with FWHM ≈19.4 μm. The simulation results for the thermoelectrical effects are in line with the experimental results observed through AFM detection of thermal expansion, showing the same distribution, and similar FWHM, as Figure 10.

## Discussion

This research characterises an active microscale optical element which can be electrically controlled. The resulting responses of the systems were investigated using two experimental approaches. First, a homodyne detection enhanced SPR setup was used to provide access to the modulation of the electric field. This method provides access to the modulation of the electric field induced by varying Joule heating. Second, the spatially resolved thermal distribution of the active plasmonic element and the surrounding environment was measured through the use of SJEM. This information is required to fully model the spatial distribution of the induced electric field changes.

While this investigation focused on the behaviour of a single active plasmonic element, the combination of high localisation and the ability to modulate individual plasmonic elements at unique frequencies enables the design of arrays of such active elements which can be operated simultaneously. This could be applied to arrays of elements whose size and pitch is below the diffraction limit of light enabling sub-diffraction-limit applications.

As before, a combination of experimentally verified simulations and direct investigations through SJEM allows various parameters to be analysed, such as the exact geometry and corresponding localisation. This aids in optimisation with the aim to further improve on physical constraints.

Both experimental methods, along with simulations, provide the basis for an optimised design of the discussed active plasmonic element giving access to a variety of possible applications. This plasmonic element was developed to be used as a key feature in a new sub-diffraction-limit imaging technique currently under development.

## Conclusion

In summary, using correlative methods to investigate a single device provides complementary information about desirable material properties intrinsic to the active plasmonic element. Only the combination of both experimental methods discussed here provides the complete set of information required for an optimised design of the element.
